# Effect of diabetes on patient-reported outcome measures at one year after laminoplasty for cervical spondylotic myelopathy

**DOI:** 10.1038/s41598-022-13838-2

**Published:** 2022-06-11

**Authors:** Kosei Nagata, Junya Miyahara, Hideki Nakamoto, Naohiro Kawamura, Yujiro Takeshita, Akiro Higashikawa, Takashi Ono, Masayoshi Fukushima, Rentaro Okazaki, Nobuhiro Hara, So Kato, Toru Doi, Yuki Taniguchi, Yoshitaka Matsubayashi, Sakae Tanaka, Yasushi Oshima

**Affiliations:** 1 Department of Orthopaedic Surgery and Spinal Surgery, The University of Tokyo, 7-3-1 Hongo, Bunkyo-ku, Tokyo 113-8655 Japan; 2 The University of Tokyo Spine Group (UTSG), Tokyo, Japan; 3 Department of Spine and Orthopedic Surgery, Japanese Red Cross Medical Center, Tokyo, Japan; 4 Department of Orthopedic Surgery, Yokohama Rosai Hospital, Kanagawa, Japan; 5 Department of Orthopedic Surgery, Kanto Rosai Hospital, Kanagawa, Japan; 6 Department of Spinal Surgery, Japan Community Health-Care Organization Tokyo Shinjuku Medical Center, Tokyo, Japan; 7 Spine Center, Toranomon Hospital, Tokyo, Japan; 8 Department of Orthopedic Surgery, Japanese Red Cross Saitama Hospital, Saitama, Japan; 9 Department of Orthopedic Surgery, Japanese Red Cross Musashino Hospital, Tokyo, Japan

**Keywords:** Neuroscience, Endocrinology, Medical research, Neurology

## Abstract

Although patients with diabetes reportedly have more peripheral neuropathy, the impacts of diabetes on postoperative recovery in pain and patient-reported outcome measures (PROMs) after laminoplasty for cervical spondylotic myelopathy (CSM) is not well characterized. The authors aimed to elucidate the effects of diabetes on neck/arm/hand/leg/foot pain and PROMs after laminoplasty CSM. The authors retrospectively reviewed 339 patients (82 with diabetes and 257 without) who underwent laminoplasty between C3 and C7 in 11 hospitals during April 2017 –October 2019. Preoperative Numerical Rating Scale (NRS) scores in all five areas, the Short Form-12 Mental Component Summary, Euro quality of life 5-dimension, Neck Disability Index, and the Core Outcome Measures Index-Neck) were comparable between the groups. The between-group differences were also not significant in NRS scores and PROMs one year after surgery. The change score of NRS hand pain was larger in the diabetic group than the nondiabetic group. The diabetic group showed worse preoperative score but greater improvement in the Short Form-12 Physical Component Summary than the nondiabetic group, following comparable score one year after surgery. These data indicated that the preoperative presence of diabetes, at least, did not adversely affect pain or PROMs one year after laminoplasty for CSM.

## Introduction

Cervical spondylotic myelopathy (CSM) is a common and increasingly observed degenerative disorder causing spinal cord compression and neurological deterioration^1^. Surgical decompression via laminoplasty is the standard treatment for CSM and can prevent the progression of neurological deficits as well as improve neurological function and quality of life^2^. Surgical outcomes should be evaluated using patient-reported outcome measures (PROMs).

Diabetic neuropathy following diabetes mellitus can profoundly impair the quality of life. Diabetes mellitus is a chronic systemic disease characterized by multiple neurologic sequelae mainly associated with diabetic neuropathy, chronic peripheral pain due to microvascular changes, and irreversible nerve damage^3–5^. The reported prevalence of painful diabetic peripheral neuropathy shows wide variability (range, 3–65%) owing to differences in sampling methods and diagnostic criteria^3–9^. It is a well-known fact that patients with diabetic neuropathy have sensory disturbances in the extremities and typically show stocking-and-glove patterns of sensory deficit^3,10^. Compromised vascularity and secondary peripheral neuropathy may affect the recovery of nerve roots even after surgical decompression^11^.

Several reports showed diabetic effects on surgical outcomes after surgery for CSM, and their results were controversial^10,12–14^. These studies showed that diabetes had equivalent effects^13,14^ or negative effects on gait, sensory, and bladder function^10^. Previous studies used JOA score, which was evaluated by doctors^10,13^. The other study was heterogenous; including patients undergoing anterior cervical spine surgery or lumbar spine surgery^12^. Therefore, the diabetic effects on PROMs and pain after cervical laminoplasty surgery have not been well characterized in patients with CSM. In the present multicenter study investigating the surgical outcomes of laminoplasty for CSM, the authors aimed to investigate the impact of diabetes on pain as well as PROMs after laminoplasty for CSM with an adequately large sample size.

## Materials and methods

### Ethics

A prospective spine surgery registry was started at eight institutions in the greater Tokyo metropolitan area, after obtaining approval from the Clinical Research Support Center of the University of Tokyo Hospital (10,335-(3)) and the institutional review boards of all participating hospitals i.e., The University of Tokyo Hospital, Japanese Red Cross Medical Center, Yokohama Rosai Hospital, Kanto Rosai Hospital, Japan Community Health-care Organization Tokyo Shinjuku Medical Center, Toranomon Hospital, Japanese Red Cross Saitama Hospital, and Japanese Red Cross Musashino Hospital. The present study was carried out in accordance with the relevant guidelines and regulations/ethical principles of the Declaration of Helsinki. The authors have obtained informed consent form with opt-out method from patients.

### Patients

The authors evaluated a consecutive cohort of patients who were diagnosed with CSM and underwent posterior cervical decompression at 11 different institutions between April 2017 and October 2019. Diagnosis was made by board-certified spine surgeons based on neurological examinations as well as MRI or myelography evaluations (existence of the effacement of subarachnoid space with spinal cord compression). The study’s inclusion criteria were as follows: symptomatic CSM (at least one clinical sign of myelopathy), surgical level between C3 and C7, evidence of cervical spinal cord compression on magnetic resonance imaging or cervical myelogram-computed tomography, and no previous cervical spine surgery. The study’s exclusion criteria were as follows: diagnosis of posterior longitudinal ligament ossification, spinal tumors, trauma, or infectious diseases; aged younger than 18 years; emergency surgery; surgery involving the thoracic spine; and fixation surgery.

### Patient data collection

Data on the clinical characteristics of the patients, including age, sex, body mass index, current smoking history, American Society of Anesthesiologists (ASA) classification, and medical history, were retrospectively collected. Preoperative laboratory data were assessed. Patients with a fasting blood glucose level of 126 mg/dL and glycated hemoglobin (HbA1c) levels of 6.5% or those previously diagnosed with diabetes by a diabetes specialist were considered to have diabetes^15^. Diabetes specialists at each hospital were able to sufficiently control the blood sugar levels of patients diagnosed with diabetes throughout the immediate perioperative period. Blood glucose levels were checked four times a day, and a sliding scale insulin coverage was adopted to avoid perioperative hyperglycemia until the patient’s food intake stabilized. Surgical factors, including operative time and estimated blood loss, were registered. Surgeons in charge were asked to report all intraoperative complications, including nerve root damage and dural tear, and postoperative complications within 30 days after surgery.

### Patient-reported outcome measures

At baseline and one year after surgery, all patients were asked to answer a booklet of questionnaires, including the Japanese version of the (1) Numerical Rating Scale (NRS), (2) the Short Form-12 Physical Component Summary (PCS), (3) Mental Component Summary (MCS), (4) Euro quality of life 5-dimmension (EQ-5D)-3L to assess health-related quality of life^16^, (5) Neck Disability Index (NDI) to assess pain-associated disability, and (6) Core Outcome Measures Index (COMI)-Neck^17^. The NRS measures the intensity of pain over the preceding 4 weeks; the scores range from 0 (no pain at all) to 10 (worst pain imaginable). To evaluate the diabetic effect on axial pain and residual radiculopathy, the authors analyzed the following five NRS domains; neck, arm, hand, leg, and foot pain. Each corresponding body part had been discussed in previous reports^18^. To determine treatment satisfaction, a 7-point Likert scale was used, wherein, the patients were asked to answer whether they were satisfied with the treatment, with possible answers of “very satisfied,” “satisfied,” “somewhat satisfied,” “neither,” “dissatisfied,” “somewhat dissatisfied,” and “very dissatisfied”^17^.

### Statistical analysis

Baseline demographic and clinical characteristics in the diabetic and nondiabetic groups were compared using the Chi-square test for categorical variables and Student’s *t*-test for continuous variables. The Student’s *t*-test was used to examine intergroup differences with respect to pre- and postoperative NRS, PCS, MCS, EQ-5D, NDI, and COMI-Neck scores. For further evaluation of the difference in each outcome score, the authors calculated the adjusted p values by inverse probability weighting method after calculating propensity scores based on seven variables (age, sex, BMI, smoking status, ASA class, operative time, and estimated blood loss) as per a previous report^12,19^. The correlations between diabetes-related factors (preoperative HbA1c level and fasting blood sugar) and postoperative outcomes was measured using Spearman’s rank correlation coefficients. We defined the correlation as the value of ρ > 0.40 with p value < 0.05. All data analyses were performed using SPSS version 21.0 statistical software (SPSS, Inc., Chicago, IL, USA).

The sample size for this study was calculated using G*Power version 3.1. With an approximately 20–25% prevalence of diabetes as reported in a previous prospectively collected database^10,13,20^, a total sample size of at least 200 patients was required with a power of > 0.8, significance level of *P* < 0.05, and effect size of < 0.2.

## Results

**Figure 1:**
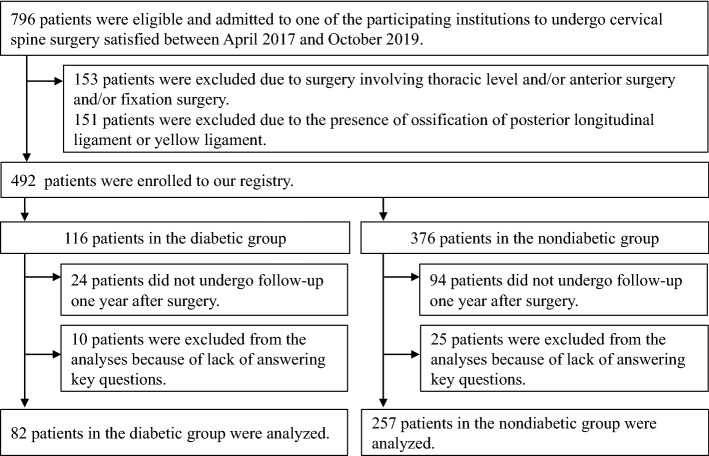
Flowchart of the study population.

Among the 339 patients who satisfied the inclusion and exclusion criteria, 82 (24.2%) and 257 (75.8%) were grouped into the diabetic and nondiabetic groups, respectively (Fig. 1). No significant intergroup differences were observed with respect to age (mean 70.1. vs. 71.0 years, *P* = 0.575), male/female ratio (58 men:24 women vs. 175 men:82 women, *P* = 0.653), and current smoking status (12.2% smokers vs. 11.1% nonsmokers, *P* = 0.454) (Table 1). The diabetic group had a higher BMI (25.2 vs. 23.7 kg/m^2^, *P* = 0.012) and included a higher percentage of patient with an ASA classification of ≥ 3 (19.5% vs. 8.0%, *P* < 0.001), hypertension (58.6% vs. 41.1%, *P* = 0.022), and hyperlipidemia (26.4% vs. 11.1%, *P* = 0.006) than the nondiabetic group. No significant intergroup differences were observed with respect to hemodialysis, rheumatoid arthritis, and past history of stroke. The diabetic group had a significantly higher mean HbA1c level (7.0% vs. 5.8%, *P* < 0.001), fasting blood sugar (161.3 vs. 112.2 mg/dL, *P* < 0.001), and creatinine (1.4 vs. 1.0 mg/dL, *P* = 0.009) than the nondiabetic group. The diabetic group had a lower mean value of total cholesterol than the nondiabetic group (183.1 vs. 200.9 mg/dL, *P* = 0.001). Although the diabetic group had a lower value of mean low density lipoprotein cholesterol than the nondiabetic group, the difference was not statistically significant (97.4 vs. 110.4 mg/dL, *P* = 0.059).

**Table 1:** Demographic and patient characteristics.

	Diabetic group	Nondiabetic group	*P* value
	(N = 82)	(N = 257)	
Age^a^ (yr)	70.1 ± 11.3	71.0 ± 11.0	0.575
Male/Female^b^	58/24	175/82	0.653
BMI^a^ (kg/m^2^)	25.2 ± 4.9	23.7 ± 3.6	0.012
Current Smoker^b^ (%)	12.2	11.1	0.454
ASA classification ≥ 3^b^ (%)	19.5	8.0	0.033
Hypertension^b^ (%)	58.6	41.1	0.022
Hyperlipidemia^b^ (%)	26.4	11.1	0.006
Hemodialysis^b^ (%)	6.1	1.9	0.053
Rheumatoid Arthritis^b^ (%)	1.2	1.9	0.664
Past history of stroke^b^ (%)	9.8	4.4	0.115
HbA1c^a^	7.0 ± 0.8	5.8 ± 0.5	< 0.001
Fasting blood sugar^a^	161.3 ± 52.2	112.2 ± 32.1	< 0.001
Creatinine^a^	1.4 ± 1.9	1.0 ± 1.0	0.009
Total Cholesterol^a^	183.1 ± 44.4	200.9 ± 34.0	0.007
Low Density Lipoprotein cholesterol^a^	97.4 ± 34.7	110.4 ± 29.0	0.059

*BMI* body mass index, *ASA* American Society of Anesthesiologists.

^a^The values are given as the mean and the standard deviation.

^b^The values are given as the percentage in each group.

No significant intergroup differences were observed with respect to operative time (139.7 vs. 136.2 min, *P* = 0.590) and estimated blood loss (102.0 vs. 98.4 mL, *P* = 0.855) (Table 2). No significant intergroup differences were observed in complications during surgery and within 30 days after surgery.

**Table 2:** Comparison of surgical factors for patients with diabetes and those without diabetes.

	Diabetic group	Nondiabetic group	*P* value
	(N = 82)	(N = 257)	
Operation time^a^ (min)	139.7 ± 45.9	136.2 ± 51.5	0.590
Estimated blood loss^a^ (ml)	102.0 ± 162.5	98.4 ± 153.7	0.855
Nerve root damage (%)	0	0	NA
Dural tear (%)	1.2	2.3	0.863
Surgical site infection (%)	3.7	2.7	0.952
Urinary tract infection (%)	2.4	0.8	0.532
Respiratory tract infection (%)	2.4	0.4	0.294

Other values are given as the percentage in each group.

^a^The values are given as the mean and the standard deviation.

The absolute changes in NRS scores are shown in Table 3. Pre- and postoperative NRS scores in neck/arm/leg/foot were comparable between the two groups (*P* > 0.05). There were no significant differences in the change NRS scores in neck/arm/leg/foot with or without adjustment by propensity score. Although pre- and postoperative NRS hand pain scores showed no significant difference between the two groups, the change score of NRS hand pain was larger in the diabetic group than the nondiabetic group (1.2 vs. 0.3, *P* = 0.034, Adjusted *P* = 0.017).

**Table 3:** Comparison of preoperative and postoperative numeric rating scales for patients with diabetes and those without diabetes.

NRSs	Outcome	Diabetic Group	Nondiabetic Group	*P* value	Adjusted *P* value
NRS neck pain	Preoperative	2.4 ± 2.9	2.8 ± 2.8	0.290	0.439
	Postoperative	2.1 ± 2.3	2.0 ± 2.3	0.909	0.986
	Change	0.3 ± 3.2	0.8 ± 2.9	0.169	0.334
NRS arm pain	Preoperative	3.1 ± 3.3	3.0 ± 3.0	0.797	0.649
	Postoperative	1.9 ± 2.6	2.3 ± 2.8	0.253	0.208
	Change	1.3 ± 3.6	0.6 ± 3.2	0.166	0.146
NRS hand pain	Preoperative	3.3 ± 3.3	3.0 ± 3.1	0.478	0.296
	Postoperative	2.0 ± 2.7	2.6 ± 2.9	0.113	0.141
	Change	1.2 ± 3.2	0.3 ± 3.3	0.034	0.017
NRS leg pain	Preoperative	2.6 ± 3.1	2.9 ± 3.0	0.505	0.565
	Postoperative	2.5 ± 2.8	2.5 ± 2.9	0.968	0.738
	Change	0.1 ± 3.3	0.3 ± 3.4	0.918	0.932
NRS foot pain	Preoperative	1.8 ± 2.9	1.9 ± 2.7	0.701	0.635
	Postoperative	1.8 ± 2.5	1.7 ± 2.7	0.809	0.587
	Change	0.0 ± 2.9	0.1 ± 2.7	0.189	0.628

*NRS* numeric rating scale.

The trends in PROMs are shown in Table 4. The diabetic group had a lower preoperative PCS score than the nondiabetic group with or without adjustment (22.0 vs. 28.9, *P* = 0.001, Adjusted *P* = 0.028), and no significant intergroup differences were observed in postoperative PCS scores (30.4 vs. 32.7, *P* = 0.324). Hence, the change score in PCS was larger in the diabetic group than the nondiabetic group, but the difference was not significant after the adjustment by the propensity score (8.4 vs. 3.7, *P* = 0.039, Adjusted *P* = 0.151). No significant intergroup differences were observed in pre- and postoperative MCS, EQ-5D, NDI, and COMI-Neck scores. There is no at least moderate correlation between diabetes-related factors (preoperative HbA1c level and fasting blood sugar) and pain/PROMs (Sup Table 1).

**Table 4:** Comparison of preoperative and postoperative patient reported outcome measures for patients with diabetes and those without diabetes.

PROMs	Outcome	Diabetic group	Nondiabetic group	*P* vlaue	Adjusted *P* value
SF-12 PCS	Preoperative	22.0 ± 15.5	28.9 ± 17.0	0.001	0.028
	Postoperative	30.4 ± 16.7	32.7 ± 16.2	0.324	0.091
	Change	8.4 ± 16.9	3.7 ± 15.3	0.039	0.151
SF-12 MCS	Preoperative	51.6 ± 11.7	48.8 ± 10.7	0.072	0.165
	Postoperative	53.4 ± 11.1	52.2 ± 9.5	0.336	0.352
	Change	2.1 ± 12.2	3.3 ± 11.5	0.185	0.396
EQ-5D	Preoperative	0.54 ± 0.27	0.56 ± 0.22	0.464	0.927
	Postoperative	0.63 ± 0.16	0.65 ± 0.21	0.536	0.727
	Change	0.09 ± 0.25	0.08 ± 0.21	0.941	0.834
NDI	Preoperative	37.7 ± 19.7	38.1 ± 20.2	0.871	0.637
	Postoperative	27.0 ± 17.5	27.4 ± 18.4	0.891	0.886
	Change	9.3 ± 18.6	10.6 ± 20.4	0.563	0.326
COMI-Neck	Preoperative	5.5 ± 2.2	5.8 ± 2.1	0.316	0.189
	Postoperative	3.5 ± 2.2	3.9 ± 2.5	0.248	0.257
	Change	1.9 ± 2.3	2.0 ± 2.7	0.692	0.493

*PROM* patient reported outcome measure, *SF-12* the Short Form-12, *PCS* physical compornent summary, *MCS* mental compornent summary, *NDI* neck disability index, *EQ-5D* Euro-quality of life-5 dimension, *COMI* core outcome measure index.

Table 5 summarizes the answers to questions on satisfaction. The diabetic group tended to have a higher proportion of patients answering “very satisfied” or “satisfied” than the nondiabetic group, although the difference was not significant (58.5% vs. 47.5%, *P* = 0.081). The diabetic group had a higher total percentage of patients who answered “very satisfied,” “satisfied,” or “somewhat satisfied” than the nondiabetic group (74.4% vs. 64.6%, *P* = 0.001).

**Table 5:** Comparison of satisfaction between the diabetic and nondiabetic group.

	Diabetic group	Nondiabetic group
	(N = 82)	(N = 257)
Very satisfied-n (%)	15 (18.3)	50 (19.5)
Satisfied-n (%)	33 (40.2)	72 (28.0)
Somewhat satisfied-n (%)	13 (15.9)	44 (17.1)
Neither-n (%)	11 (13.4)	59 (23.0)
Dissatisfied-n (%)	3 (3.7)	9 (3.5)
Somewhat dissatisfied-n (%)	4 (4.9)	13 (5.1)
Very dissatisfied-n (%)	3 (3.7)	10 (3.9)

## Discussion

To the best of our knowledge, this has been the largest study that investigated the effects of diabetes on multiple NRS scores with PROMs following elective laminoplasty for CSM. In the present study, pre- and postoperative NRS scores for neck/arm/hand/leg/foot pain were comparable between the two groups. The diabetic group showed greater improvement in NRS hand scores than the nondiabetic group. No significant intergroup differences in pre- and postoperative MCS, EQ-5D, NDI, and COMI-Neck scores were noted. Although the diabetic group had lower preoperative PCS scores than the nondiabetic group, the postoperative PCS scores of the two groups were comparable. Moreover, patients in the diabetic group tended to be satisfied with the surgical results. Collectively, these data indicated that the presence of diabetes did not, at least, adversely affect surgical outcomes of laminoplasty for CSM.

The present study showed that the diabetic group had higher prevalence rates of ASA classification ≥ 3, consistent with the findings of a previous study^13^. Although the diabetic group tended to have more risk factors, the current study revealed no intergroup differences with respect to perioperative complications. Strict glycemic control may play an important role. The mean and maximum HbA1c level was 7.0% and 8.9%, respectively, even in the diabetic group. This relatively strict glycemic control enforced by diabetes specialists before and after laminoplasty surgery may have contributed to the lack of intergroup differences not only in complications but also in the non-inferiority in pain control. Appropriate perioperative blood glucose control is associated with a reasonable recovery after cervical decompression^10^ or belief in self-efficacy and active behavior^21^. Several trials have shown significant improvements in peripheral nerve function with intensive glucose control^22^.

Only a few large-scale reports have investigated the effects of diabetes on laminoplasty using PROMs^12^. In the present study, the diabetic and nondiabetic groups showed similar pre- and postoperative MCS/EQ-5D/NDI/COMI-Neck scores, except for PCS. Diabetes has been reported as a risk factor for neck and back pain^23,24^. Armaghani et al*.* argued that patients with diabetes showed minor improvements in the NDI or Oswestry Disability Index and EQ-5D scores compared with patients without diabetes^12^. They analyzed patients undergoing back surgery and those undergoing neck surgery together^12^ and thereby making interpretation difficult. Alternatively, Arnold et al*.* found that patients with diabetes did not differ from those without diabetes in terms of pre- and postoperative NDI^14^ and Nori et al*.* argued that diabetes did not negatively affect neck pain after posterior cervical decompression^13^. The present study was consistent with these reports^13,14^ in that diabetic patients had comparable postoperative outcomes and we showed this point by pain-associate PROMs. The effects of diabetes may differ between patient with back and neck pain. Given that diabetes impairs bone fusion, the effects of diabetes can be greater following posterior lumbar interbody fusion surgeries^25^, which contributes to mechanical instability and the occurrence of back pain^13^. In contrast, considering that cervical laminoplasty is not performed for the purpose of bone fusion, the effects of diabetes can be minor. These mechanisms can explain why no significant differences were observed for neck pain-associated outcome measures, including NRS neck pain, EQ-5D, NDI, and multidimensional COMI-Neck, in the current study.

In the present study, the diabetic group showed lower preoperative PCS scores than the nondiabetic group; however, the two groups had comparable postoperative PCS scores. This finding differed from that reported by Arnold *et al*.^14^; they reported no significant intergroup differences in preoperative PCS, but patients with diabetes experienced significantly lesser improvement than those without diabetes^14^. Our cohort had an average age of 70.8 years, while Arnold’s cohort had an average age of 56.4 years^14^. The relatively older our cohort with low preoperative PCS scores may have affected the difference. Because of the relatively low reliability of PCS for evaluation of health transition among patients aged 75 or over^26^, multiple PROMs should be used when the average age of the cohort is high. Low preoperative PCS scores can be associated with lower leg strength and proprioception^19^, which is greater risk of preoperative slower walking speed, especially in the elderly^27,28^. The slower walking speed due to diabetic neuropathy could mimic walking impairment due to CSM, resulting in early surgical intervention in the diabetic group. Early surgical intervention is an important factor for good clinical outcomes^29^ and can explain why the diabetic group in the present study had comparable postoperative PCS scores, despite having lower preoperative PCS scores. To prove this hypothesis, further large size studies linking duration of CSM symptoms and multiple PROMs are warranted.

The authors believe that this study adds to the contemporary knowledge related to the effect of diabetes on patients undergoing cervical posterior decompression surgery. However, some limitations of our study should be considered when interpreting our results. First, the current study did not analyze detailed information for diabetes except for preoperative HbA1c level. We were unable to collect detailed information on diabetes, including duration, medication history, or trend of HbA1c. Second, the follow-up duration was one year, not two years after surgery, although PROMs assessed at one year adequately predict long-term (24-month) outcomes^30^. Third, double door or open door laminoplasty was determined by the surgeon in charge at each participating hospital due to the multicenter study design. In addition, relatively elder cohorts may undergo laminoplasty for the purpose myelopathy rather than pain caused by radiculopathy and the effect of laminoplasty on peripheral may be limited.

## Conclusions

The results of the present multicenter cohort study showed that diabetes did not adversely affect improvement in pain and PROMs one year after laminoplasty for CSM, although the diabetic group had lower preoperative PCS scores than the nondiabetic group. Under the strict glycemic control before and after surgery, surgeons can use this information when counseling patients who have CSM with diabetes about the expected outcomes of laminoplasty.

## Supplementary Information

Supplementary Table 1.
