# Supplementary material for: Effect of diabetes on patient-reported outcome measures at one year after laminoplasty for cervical spondylotic myelopathy
Source: Sci Rep. 2022 Jun 11;12:9684. doi: 10.1038/s41598-022-13838-2 (PMC9188556; doi:10.1038/s41598-022-13838-2)
Supplement: Supplementary file 1 — Supplementary Table 1. [file 41598_2022_13838_MOESM1_ESM.docx]

| **Sup Table 1.** Spearman’s rank correlation coefficients (ρ value) | | | |
| --- | --- | --- | --- |
| Outcome |  | HbA1c | Fasting blood sugar |
| NRS Neck pain | Preoperative | -.132^*^ | -.200** |
|  | Postoperative | -0.044 | -0.041 |
|  | Change | -0.044 | 0.030 |
| NRS Arm pain | Preoperative | -0.048 | -0.059 |
|  | Postoperative | -0.048 | -0.020 |
|  | Change | -.128^*^ | -.194** |
| NRS Hand pain | Preoperative | -0.059 | -0.065 |
|  | Postoperative | -0.042 | -0.033 |
|  | Change | 0.024 | -0.070 |
| NRS Leg pain | Preoperative | -0.06 | -0.081 |
|  | Postoperative | -0.044 | -0.074 |
|  | Change | -0.022 | -.141* |
| NRS Foot pain | Preoperative | -0.063 | 0.030 |
|  | Postoperative | 0.003 | 0.042 |
|  | Change | 0.023 | -0.106 |
| SF-12 PCS | Preoperative | -0.103 | -0.051 |
|  | Postoperative | -0.033 | -.117* |
|  | Change | 0.028 | -0.028 |
| SF-12 MCS | Preoperative | 0.114 | 0.025 |
|  | Postoperative | -0.055 | -0.044 |
|  | Change | 0.064 | -.116* |
| EQ-5D | Preoperative | 0.011 | -0.009 |
|  | Postoperative | 0.038 | -0.024 |
|  | Change | -0.063 | 0.009 |
| NDI | Preoperative | -0.034 | 0.025 |
|  | Postoperative | 0.01 | -0.094 |
|  | Change | -0.017 | -0.102 |
| COMI-Neck | Preoperative | -0.033 | -0.028 |
|  | Postoperative | -0.043 | 0.089 |
|  | Change | -0.007 | 0.082 |
| * P value <0.05, ** P value <0.01 NRS, Numeric Rating Scale; SF-12, the Short Form-12; PCS, Physical compornent summary; MCS, Mental compornent summary; NDI, Neck Disability Index; EQ-5D, Euro-quality of life-5 dimension; COMI, Core outcome measure index. | | | |
